# Factors associated with intentions to adhere to colorectal cancer screening follow-up exams

**DOI:** 10.1186/1471-2458-6-272

**Published:** 2006-11-06

**Authors:** Ying-Fang Zheng, Tami Saito, Miyako Takahashi, Teruo Ishibashi, Ichiro Kai

**Affiliations:** 1 Department of Social Gerontology, School of Health Sciences and Nursing, Graduate School of Medicine, University of Tokyo, 7-3-1 Hongo, Bunkyo-ku, Tokyo 113-0033 Japan; 2 Ishibashi Occupational Safety and Health Consultant Office, 337-7 Saku City, Nagano 385-0026 Japan

## Abstract

**Background:**

To increase adherence rate to recommendations for follow-up after abnormal colorectal cancer (CRC) screening results, factors that inhibit and facilitate follow-up must be identified. The purpose of this study was to identify the factors associated with intention to adhere to CRC screening follow-up exams.

**Methods:**

During a 4-week period in October 2003, this survey was conducted with 426 subjects participating in a community-based CRC screening program in Nagano, Japan. Study measures included intention to adhere to recommendation for clinical follow-up in the event of an abnormal fecal occult blood test (FOBT) result, perceived susceptibility and severity of CRC, perceived benefits and barriers related to undergoing follow-up examination, social support, knowledge of CRC risk factors, health status, previous CRC screening, personality and social demographic characteristics. Univariate and multivariate logistic regression analyses on intention to adhere to recommendations for follow-up were performed.

**Results:**

Among the 288 individuals analyzed, approximately 74.7% indicated that they would definitely adhere to recommendations for follow-up. After controlling for age, gender, marital status, education, economic status, trait anxiety, bowel symptoms, family history of CRC, and previous screening FOBT, analyses revealed that lower levels of perceived barriers, higher levers of perceived benefits and knowledge of CRC risk factors were significantly associated with high intention respectively.

**Conclusion:**

The results of this study suggest that future interventions should focus on reducing modifiable barriers by clarifying misperceptions about follow-up, promoting the acceptance of complete diagnostic evaluations, addressing psychological distress, and making follow-up testing more convenient and accessible. Moreover, educating the public regarding the risk factors of CRC and increasing understanding of the benefits of follow-up is also important.

## Background

The incidence and mortality rates of colorectal cancer (CRC) have increased markedly in Japan. Age-adjusted death rates of CRC have doubled during the past few decades, from 8.6 and 7.5 per 100,000 males and females respectively, in 1950 to 22.8 to 13.5 per 100,000 in 2003 [1]. 5-year survival rates for CRC vary dramatically according to the stage of detection from 25% when there is distant spread of the disease, to 95% when the disease is localized [2]. Thus, the prevention and early detection of CRC is of great public health importance.

Screening using the fecal occult blood test (FOBT) has been shown to reduce the incidence and mortality of CRC in randomized clinical trials [3]. In addition, results from case-controlled studies have suggested that screening using immunochemical FOBT could reduce mortality from CRC by 60% or more for Japanese populations aged 40 year and older who screen annually [4]. Patients with an abnormal FOBT (FOBT+) in these trials were routinely followed up with a complete diagnostic evaluation (CDE), and the reduction in mortality demonstrated in FOBT screening trials can be attributed to the use of follow-up CDE. According to the Japanese Ministry of Health and Welfare's CRC screening guidelines, CDE includes colonoscopy or the combination of a double air contrast barium enema X-ray and flexible sigmoidoscopy, or in cases where an endoscopy cannot be completed, a double-contrast barium enema examination is also temporarily acceptable [5].

Under the auspices of the Health and Medical Service Law for the Aged, a national CRC screening program was initiated in Japan in 1992 as part of a public health policy. Asymptomatic populations over the age of 40 are recommended to participate in the screening program, which uses a 2-day immunochemical FOBT. Despite strong consensus from public health academics and cancer epidemiologists in supporting CRC screening, fewer than 60% of screening FOBT+ patients received follow-up evaluation, and this poor follow-up rate remained unchanged between 1992 and 2003 [6]. Limited follow-up rates pose an important obstacle to achieving overall CRC screening effectiveness. Moreover, non-adherence to CDE has been implicated as contributing to adverse outcomes in retrospective analysis of advanced CRC [7]. It remains a matter of urgency to improve clinical follow-up compliance. Therefore, to assist in the development of effective interventions, studies which provide knowledge regarding the factors that facilitate or impede clinical follow up positive FOBT results are needed.

Inadequate follow-up of FOBT+ patients have also been reported in the United States [8-13]. Recently, Nadel and colleagues have showed that 31.6% of the FOBT+ patients did not have any follow-up exams. Previous studies have focused on physicians' potential barriers and facilitating factors implicated in the provision of follow-up exams [10-16], including physicians' background (e.g., board certification, time in practice, specialty, etc.), cognitive and psychosocial representations (e.g., perceived CDE effectiveness, belief that CDE is standard practice, intention to evaluate a FOBT+ with CDE, concerns about CDE related cost, etc.), practice environment (e.g., number of physicians, etc.) and patient characteristics. Regarding patient characteristics, being a male [12], having high social class [16], with the greater number of positive Hemoccult windows, having family history of CRC, patients' CRC worry [10], and consultation with a gastroenterologist [8] have been reported to improve physicians' chances to order an adequate follow-up evaluation.

Non-adherence is perceived to be a global problem, in that both providers and patients appear to share responsibility for the problem [11,14,16,17]. Very little, if any, few published studies focus on factors impacting on FOBT+ patients' decisions to undergo follow-up examinations. Previous research has suggested that noncompliant FOBT+ patients reported time restraints and an absence of current symptoms as major reasons for failure to undergo clinical follow-up after a positive FOBT [18]. However, it must be noted that the study sample employed was small and that the research methodology used descriptive methods only. Recently, Tashiro reported that in comparison with patients who complied with follow-up recommendations, noncompliant patients who were 70 years and older demonstrated worse mental health status and physical functioning [19]. However this study included a methodological limitation in that it measured the FOBT+ patients' quality of life only. In order to target patient education and intervention strategies to improve quality of care for patients with abnormal FOBT, and ultimately to decrease non-compliance to clinical follow-up recommendations, it is important to understand the characteristics motivating and inhibiting patients compliance with clinical follow-up recommendations.

**Figure 1:**
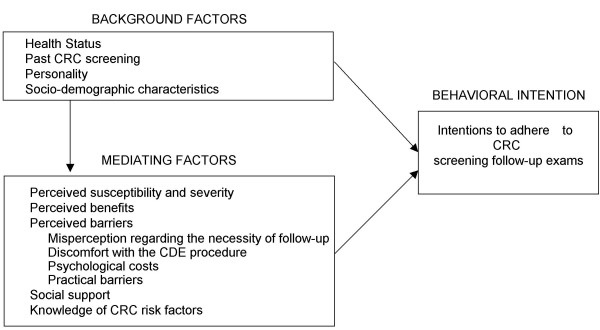
Conceptualization of factors associated with intentions to adhere to CRC screening follow-up exams.

Conceptual framework (Fig.1)

In order to contribute to intervention design, this study examined the factors that may impact intention to comply with clinical follow-up recommendations. We incorporated elements of the Health Belief Model (HBM) [20,21] and the Theory of Planned Behavior [22] to direct the present research. According to the Theory of Planned Behavior, one of the strongest immediate determinants of behavior is a person's intention to perform it. Empirically, intention to undergo screening remains one of the strongest and most consistent factors associated with actual cancer preventive behavior, including breast [23,24], prostate [25], cervical [24], and colon [26,27] cancer screening. The main outcome of variable of this study was intention.

The present study used four original constructs from HBM as identifying factors that may facilitate or restrain follow-up intention. According to the original HBM, a person's decision regarding undertaking a preventive action related to a disease is influenced primarily by the following four beliefs: (a) perceived susceptibility (one's opinion of the chances of getting a condition), (b) perceived severity (one's opinion of how serious a condition and its sequelae are), (c) perceived barriers (one's opinion of the tangible and psychological costs of the advised action), and (d) perceived benefits (one's opinion of the efficacy of the advised action to reduce risk of seriousness of impact). Furthermore, knowledge regarding cancer [28-31] and social support from family and/or the health care professionals [32-37] that have been shown to be related or associated with screening behavior and intention were also included in our framework.

## Methods

### Setting

The study was conducted in S City in the Nagano Prefecture located in the center of Japan, northwest of Tokyo. The city has a population of approximately 67,800. FOBT screening is conducted at the A public hospital of S City throughout the year. Once a year in March, the city office distributes information regarding CRC screening to eligible inhabitants using a pre-paid pre-addressed postcard. Applicants select the day that suits them, and return the postcard to the City Health Department. Two weeks before the screening date, FOBT kits are mailed to perspective participants. Kits also contain printed instructions for specimen collection and applicator sticks. Screening participants are required to conduct the specimen collection at home and to return completed kits to A public hospital on the day of screening when participants will undergo consultation with a physician.

Participants are notified of test results by written correspondence 2 weeks later after undertaking the test. Participants testing positive are informed that their FOBT tested positive and that they should undergo further testing. In order to ensure follow-up for FOBT+ participants, two kinds of forms are enclosed with this letter. The first is a letter of introduction for clinical examination to be given to the medical institution. The other sheet requests permission for the hospital where the patient receives follow-up examination to forward on the results to S City Health Department. If the follow-up related information is not received within three months, public health nurses of the Health Department contact patients to encourage follow-up adherence by letter and telephone. Based on administrative data, approximately 4,320 people undertook FOBT screening in 2002 and of these 347 patients were found to have abnormal test results, of which 227 (65.4%) received further clinical evaluation.

### Procedure

Participants for this study were CRC screening participants recruited at the screening department of A public hospital. We handed letters requesting participation in the study to all participants attending the hospital for screening, after obtaining oral consent to participate in the study, willing respondents were asked to complete the anonymous questionnaire in the waiting room before consulting with the physician. Respondents were provided with pencils and clipboards to fill out the questionnaire, which required approximately 20 minutes to complete on average. The data collection covered a 4-week period in October 2003. The total number of CRC screening participants in the study period was 426.

In order to help respondents distinguish CRC screening exams options, the survey provided descriptions regarding the methods of CDE and the rate of FOBT+ results before undertaking the survey. To avoid influencing respondents' perceptions [38], we used the standard letter recommending follow-up and an information brochure about CRC screening, both of these are currently used in Japanese community-based cancer screening program, as routine procedure for distributing information. These information materials showed and explained the procedures, however did not include information regarding the benefits, costs, potential uncertainties and limitations of each test, therefore we also decided not to offer those information.

### Ethical considerations

During this study period, the University of Tokyo did not require ethical approval for anonymous questionnaire studies. After reviewing from the A public hospital and the Health Department of S City, the written authorization to conduct this study was obtained from the Mayor. Care was taken to minimize the burden on participating patients in terms of physical disruption and emotional disturbance. Participants were informed of their freedom to participate or to withdraw from the research and efforts were made to maintain privacy.

### Subjects

A total of 405 participants indicated a willingness to participate in the study. Those who declined participation gave reasons such as being uninterested (n = 8) and feeling unwell (n = 13). No data are available concerning on non-responders.

### Development of the study questionnaire

Survey development was based on the review of literatures and the researchers' research and clinical experiences as general hospital interns.

#### Dependent variables

This study employed the concept of behavioral intent as a dependent variable. We assessed intention to adhere to the recommendation for follow-up consultation in the event of an abnormal FOBT result in terms of response to the following scenario presented in the questionnaire, which states: "Today, you undertook a FOBT screen test, and you will subsequently be given a result that indicates a normal or an abnormal result. If you are informed that you had a positive FOBT and that you should go to hospital for further testing, how likely is it that you will do this?". The response categories used a five-point Likert-type scale ranging from definitely (5); probably (4); not sure (3); probably not (2); to, definitely not (1). Responses were dichotomized as 5 versus the responses less then 5. Respondents with a score of 5 were classified as having strong intention to undertake the recommended follow-up in the event of an abnormal FOBT result and were compared with respondents scoring < 5, who were classed as having weaker intention. Use of these categories is consistent with the approach suggested by Manski [39] who recommended that intention should describe the strongest probability of behavior completion. This categorization has been used in previous studies looking at intentions regarding undertaking CRC screening [27,33], and prostate cancer screening [34].

#### Predictor variables

This study used items adapted from Jacobs' revised Champion's Health Belief Model Scales [40] modifying them to measure perceived susceptibility and severity of CRC, and perceived benefits of undergoing follow-up exams. The subscales were translated using a back-translation technique. Six bilingual health professionals in the researchers' health education department conducted the translation into Japanese, ascertained the subscales' content validity, and determined the cultural appropriateness of the tool. A different bilingual individual then back translated the Japanese version into English. Relevance and accuracy were checked by a double translation and subsequent comparison of two English versions. All items for these subscales consisted of belief statements with a 5-point Likert scale ranging from strongly disagree (1) to strongly agree (5). Before the study, the test-retest reliability of the translated survey was pre-tested in 40 subjects with a 2 – 3 week interval between tests. The test-retest correlations ranged from r = 0.73 to 0.89.

##### Perceived susceptibility

Perceived susceptibility was assessed to rate one's chance of getting CRC. With respect to absolute risk, two items were asked to the respondents 'how likely are you to get CRC in the next few years and in lifetime?' For comparative risk, one item was asked respondents 'compared with other people of your age, how likely are you to get CRC in the lifetime?' Higher scores indicate a higher perceived risk of developing CRC, internal reliability in the present sample was α = 0.85.

##### Perceived severity

Participants' beliefs regarding the severity of CRC were measured by 6 items. Items assessed how severely CRC would disrupt personal health, emotional well-being, and overall severity of the health consequences. Higher scores indicate higher perceived severity of CRC, internal reliability in the present sample was α = 0.83.

##### Perceived benefits

The words "have regular check-ups to detect colon cancer" were changed to "undergo follow-up examination to detect CRC", and 6 items were included in this revised subscale. Items included concerned respondent's beliefs regarding the follow-up examination's effectiveness and attributes: finding CRC and/or polyps early, decreasing the chances of dying from CRC, and freedom from worry about CRC. Higher scores indicate a higher perceived benefit of CDE, and internal reliability in the present sample was α = 0.88.

##### Perceived barriers

Perceived barriers were measured using 11 items (Table 2). The constructs of perceived barriers to follow-up examination were developed by an extensive review of the literature on CRC endoscopic screening specifically [28,30,31,36,41-51], as well as follow-up of abnormal cervical [35,52,53], prostate [54] and breast cancer screening [55-57]. We estimated four domains of barriers; misperception regarding the necessity of follow-up (n = 2), discomfort with the CDE procedure (n = 4), psychological costs (n = 2), and practical barriers (n = 3). Respondents were asked to indicate the extent to which they strongly disagree (1) or strongly agree (5) with each of the items. Since the various potential barriers were not relative [21], these item sets did not form reliable scales according to scaling criteria, and analysis was conducted on the individual items.

##### Social support

Social support was investigated using the following questions: (1) "If you had a health problem would you be able to talk about it with family members"; and (2) "Would you be able to talk about the health problem with health care professionals easily?" Responses were recorded as yes (1) and no (0).

##### Knowledge of CRC risk factors

Items were modified from previous studies [31,58,59]. Participants were asked, "Do you know if the following things increase a person's chance of getting CRC?" Response choices included: (1) increasing age; (2) having a blood relative with bowel cancer; (3) low-fiber diet; (4) high-fat diet. The correct answer was scored as 1 and incorrect responses and missing data were scored as 0. Knowledge of risk factors was assessed after assessing perceived susceptibility.

##### Health status

Several measures of health status were used in this study. (1) Family history of bowel cancer: respondents were asked if any of their first-degree blood relatives had experienced CRC. (2) Respondents were asked if they had experienced any of the following bowel symptoms: constipation, use of laxatives, diarrhea, wind, pain in the abdomen, incontinence, blood, hemorrhoids, indigestion, and anal soreness. Each was rated for frequency during the past year (non-existent, occasionally, frequently). Responses were dichotomized as "having at least one symptom occasionally or frequently = 1" and "not = 0" for analysis. In addition, subjective health status, the existence of chronic conditions and bowel disease history were also asked.

##### Past CRC screening

Subjects were asked if they had ever undertaken FOBT, when they had been tested, if their FOBT had ever been abnormal, if follow-up had ever been recommended, or if they had ever had undergone a previous CDE. In addition, respondents were asked if they had received a CDE diagnosis outside of regular CRC screening.

##### Personality

Only the 20-item subscale of Japanese standardized Trait Anxiety Inventory was used in this study [60,61]. Internal reliability in the present sample was α = 0.86.

##### Socio-demographic characteristics

Demographic information was sought including age, gender, marital status, living arrangement, education (level of school completed), employment status, and subjective economic status.

### Data analysis

The experience of having had abnormal cancer screen results may influence patients' beliefs, knowledge, and compliance regarding future cancer screening [30,50,62]. In order to determine follow-up intention, it was deemed inappropriate to analyze persons who had and had not experienced previous follow up together. Therefore those participants who indicated that they had previously received bowel examination (e.g., CDE as well as flexible sigmoidoscopy or barium enema X-ray only) (n = 88), and those who were recommended but did not adhere to follow-up tests (n = 9) were not included in the analyses. Furthermore, individuals with a personal history of CRC (n = 4) and respondents with insufficient data on key variables on intention (n = 16) were excluded. Thus, the remaining 288 subjects formed the group focused on by this study.

Descriptive statistical data were used to summarize participant characteristics. Initially, logistic regressions were conducted to assess the independent effect of each study variable's association with follow-up intention. Furthermore, multivariate logistic regression analyses were conducted using age, gender, marital status, education, self-rated economic status, trait anxiety, bowel symptoms, family history of CRC and past FOBT screening as control variables, the social and psychosocial variables (e.g., perceived barriers, perceived benefits, perceived susceptibility, perceived severity, knowledge of CRC risk factors, and social support) were entered individually into the model to determine whether they had significant association with intention. The unadjusted and adjusted odds ratio described the association of each psychosocial variable and intention respectively. The statistical package SPSS (Version 11.5J) was used for the analysis. An alpha level of 0.05 was used to determine the statistical significance for all analyses, and all *p *values were assessed using two-sided tests.

## Results

### Characteristics of study participants (Table 1)

Information regarding the 288 subjects in the study is presented in Table 1. Approximately one quarter of subjects (23.8%) were less than 50 years of age and 20.2% were greater than 70 years of age. Due to the fact that employed workers can receive cancer screening through occupational health insurance, only one third of subjects were men (33.0%). Most subjects were currently married (89.0%) and living with a family member (94.2%). The majority did not have education beyond high school (72.4%). Approximately 60.8% were employed on a full time or a part-time basis. Approximately two-thirds indicated that their economic status was average.

The mean score on the Trait Anxiety subscale was 43.9 (8.7). Approximately one half of the sample rated their general health as fair, 31.6% as good or very good, approximately 44.8% reported having chronic disease. Approximately 55.3% had at least one bowel symptom, but none of the respondents reported having ever been diagnosed with any of the bowel diseases previously listed. The percentage of respondents who had one or more first-degree relatives with bower cancer was 14.2%. Approximately 69.3% had previously undertaken at least one FOBT screening test.

### Social-psychosocial profiles of study participants (Table 2)

#### Perceived barriers

The score of the barriers ranged from 1.4 (0.9) to 3.8 (1.3). In order to shed light on the nature of barriers to explore these factors in more detail, we analyzed the percentage of respondents who "strongly agreed or agreed" with each item. Regarding misperceptions, "unnecessary unless symptomatic" (11.4%), and "unnecessary for my age" (8.9%) were also reported in low ratios. A cluster of CDE procedure related discomfort barriers were commonly cited as follows, in order of preference: concerns about "bowel preparation" (70.9%), "pain" (63.6%), "discomfort" (64.5%), and "embarrassment" (39.6%). Regarding psychological costs, if notified of an abnormal FOBT result, 62.7% of the sample reported that they were afraid that the follow-up check would find cancer, and 37.5% of the sample identified discomfort to talk about CRC. Moreover, responses to practical barriers were low including "too busy" (9.3%), "cost" (8.5%), and "having other important things to do" (3.6%).

#### Perceived benefits

Six items measured the benefits of undertaking follow-up tests and the average score was high 25.0 (5.5) with respondents universally agreeing with most of the listed benefits: "will help find polyps" (89.8%), "will help find CRC early" (86.3%), "reduce uncertainty" (83.9%), "relief from fear of getting CRC" (72.4%), and "decrease the chance of dying from CRC" (75.7%). The lowest percentage of agreement was the item "having CDE decreases my chances of requiring radical or disfiguring surgery" (59.4%).

#### Perceived susceptibility

The average susceptibility score was 6.3 (2.7). A minority of respondents (8.9%) reported that "my risk is higher than other people of my age" and 14.5% reported, "It is possible that I could get CRC". Respondents with family history of CRC were more likely to rate them at higher risk (p < 0.001), although most still rated their risk of bowel cancer as average or even below average.

#### Perceived severity

The average seriousness score was 19.0 (5.9). The majority of respondents (68.3%) indicated that "even if CRC were detected the sequelae would last a long time", and 53.2% agreed that "the thought of getting CRC scares me". Women perceived CRC as more severe than men (p = 0.001). Furthermore, having bowel symptoms were associated with high levels of perceived seriousness (p = 0.001).

#### Knowledge of CRC risk factors

Approximately 45.8% of respondents recalled older age as a risk factor for CRC. Family history of CRC was mentioned by 77.1%. Lifestyle factors were recalled with greater frequency (low fiber diet = 91.5%; high fat diet = 85.6%). Approximately one third (29.9%) of respondents were able to recall all four risk factors for CRC.

#### Social support

More than one half (53.1%) of all subjects indicated that family members were likely to support them in the event of illness, and 55.9% of subjects said that they could communicate about the problem with health care professionals easily.

### Intention to adhere to follow-up recommendation (Table 2)

The majority (74.7%) of respondents expressed high intention, i.e., indicated that they would definitely adhere to follow-up recommendations after a hypothetical abnormal CRC screening result. None of the socio-demographic variables, health status related variables or trait anxiety was significant predictors of intention (data no shown). After controlling for age, gender, martial status, education, self-related economic status, trait anxiety, bowel symptoms, family history of CRC and past screening FOBT, the multivariate logistic regression analyses revealed that lower perceived barriers, higher perceived benefits and knowledge of CRC risk factors were significantly associated with intention separately. However, perceived susceptibility, perceived severity and social support were not statistically associated with intention in bivariate and multivariate analyses.

## Discussion

To the best of our knowledge, the present study is the first investigation into the factors relating to adherence to recommendations for clinical follow-up after an abnormal screening FOBT result. This theory-driven research presents useful findings that have not been previously investigated. Among the 288 individuals analyzed, 74.7% indicated that they would definitely adhere to recommendations for follow-up, we identified that higher perceived barriers were significantly associated with lower intention, and that high-perceived benefits and greater knowledge of CRC risk were associated with increased intention. In the following discussion we discussed in accordance with Table 2.

Several studies published to date have widely used HBM or part of HBM to design theoretical framework to facilitate understanding of CRC screening interests [31,33,36,37,46,51] and behaviors [28,30,42-44]. During the past decade, HBM has also been used to investigate Japanese stomach cancer screening behavior [62] and preventive health behaviors [32]. Despite variations in study design and measurement of cancer screening attitudes and behaviors, considerable support for the HBM has been documented. In this study, perceived barriers and perceived benefits were found to significantly associate with intention, our findings suggest that the HBM provide a useful framework for understanding patients' attitudes and beliefs regarding follow-up for abnormal CRC screening results.

Barriers to performing recommended behaviors are one of the key components in the HBM and have been shown to be strong predictors of taking action [21,63]. We assessed the four aspects of barriers, and our findings will help clinician and researchers to develop effective interventions to increase patients' compliance with recommendations for further diagnostic follow-up.

The misconception held by respondents that follow-up consultations were "unnecessary unless symptomatic" and "unnecessary for my age" reflect lack of adequate understanding regarding the purpose of screening to detect CRC or polyps at an asymptomatic stage [42]. Respondents may lack understanding of the meaning of positive FOBT results [35,64], misinterpret recommendation [35], or lack of distinction regarding asymptomatic screening versus diagnostic testing [65]. In addition, we suggested that age accounted for nonparticipation in the following two ways: (1) that the young may perceive that only older people need follow-up examination, and (2) the older may believe that they are too old and too close to death to look for illness [66]. Based on previous studies, lack of understanding of the necessity for follow-up tests were also found among participants of breast [55] and cervical cancer screening studies [35,52,53], and noncompliance was attributed to lack of adequate communication regarding screening results [35] and necessity for follow-up testing [64]. Misperception may lead some patients to belief findings are normal and do not require further attention [17]. Although follow-up guidelines for abnormal FOBT have greater consensus and less variation, Japanese cancer screening programs currently use a generic letter providing information regarding screening results and notification for follow-up. Since a well-informed screening participant may be more likely to follow up promptly after an abnormal screening result [57], improving communication of the meaning of positive screening results, the purpose and necessity of follow-up examination, and clarifying misperceptions regarding follow-up may be important components for further interventions.

Perceived unpleasantness regarding preparation for CDE and discomfort of CDE procedures have also been identified as barriers to compliance with endoscopic screening [28,30,31,36,37,41,42,44,45,47,48,50,67]. Despite lack of experience, the majority of subjects thought that procedure of CDE was uncomfortable. Results from the current sample are partially inconsistent with the literature, in that we observe significant association of perceived embarrassment and intention. It may be that since these patients already understood that CDE is invasive and unpleasant, they had already anticipated a certain degree of pain or discomfort but believed they could cope. In fact, patient who had undergone endoscopy examination found that the procedure more comfortable than expected [68,69]. Nevertheless, combined with effects to improve technical skills making CDE comfortable, health providers should assist patients in accepting the CDE [47].

If notified of abnormal FOBT results, a very high percentage of respondents perceived that they were "afraid of finding cancer", and after controlling co-variables, the perception of "uncomfortable talking about CRC" showed a negative association with intention. The psychological impact of abnormal cancer screening results, such as fear of diagnostic test, anxiety and worry about cancer were indicated in the literature regarding follow-up of cervical [53,70,71], breast [56,57,72] and prostate cancer screening [54]. Abnormal cancer screening findings and recalls for further investigation have the potential to generate psychological distress, and may be one reason of patients fail to undergo follow-up testing. Our findings indicate that the recommendation letter and the communication regarding follow-up should be created cautiously; as such communication will elicit and address patients' concern. Patients with special concerns or questions should be encouraged to contact the screening provider.

As well as findings in the FOBT and/or endoscopy screening for CRC [31,36,41,47,65,73], respondents identified time constraints such as "being busy" or "having other priorities" to undergo follow-up check. Compared with FOBT screening, follow-up employing CDE is much more demanding and invasive. Motivation and time is required for proper bowel preparation, and undertaking the test usually necessitates time off work for the participant. In Japan there are local variations in the capacity of conducting endoscopic diagnostic examinations for positive FOBT [74], difficulties in obtaining consultation and long waiting periods for follow-up medical appointments have interdependent effects on the risk for noncompliance [75], thus shortening the interval from screening to diagnostic examination, making follow-up testing more convenient and accessible may help to motivate those persons to overcome competing demands [52,55,73]. A few case studies have suggested that diagnostic examination in CRC screening were improved by reducing inconvenience barriers [75], the intervention efficiency requires further evaluation more thoroughly using studies with a randomized controlled design. Our study also identified that the necessity to pay out-of-pocket patients' costs constitutes a barrier to intention. Fees for screening are partially met by the local government, and fees for follow-up tests are partially subsidized by patients' health insurance. For example, individual payment for FOBT screening is only about 800 yen (approximately US$ 7) and individual payment for colonoscopy is approximately 7,500 yen (approximately US$ 65), with other costs (e.g., colonoscopic polypectomies) running to approximately 24,000 yen (approximately US$ 209). Previous research suggested that being uninsured or underinsured was associated with delayed or incomplete follow-up of cervical cancer screening[17] and out-of pocket costs have influenced patients' decision making [76], thus factors in willingness to pay in encouraging follow-up for abnormal FOBT screening is needed in the future.

In this study, patients with high-perception of benefits were more likely to state that they intended to adhere to follow-up recommendations than patients having low-perception of benefits. These findings support a substantial body of research on the association between benefits and intentions in participating in cancer screening [36,41]. In relation to a review of previous studies that used constructs from the HBM, Janz and Becker indicated that perceived benefits was a more important factor for sick-role behaviors than for preventive health behavior [63]. Follow-up examination appears to function more as an early detection or sick-role behavior, where anticipated benefits emerge as the stronger influencing factor [43]. Our findings can readily be interpreted within this context. Although CRC screening has convincing evidence regarding effectiveness, it appear that messages regarding CRC screening exaggerate its potential benefits and fail to mention the potential uncertainties (e.g., false-positive results of FOBT) and limitations of CRC screening (e.g., false-negative results of FOBT and CDE), as well as the possible complications associated with CDE (e.g., occasional risk of perforation, bleeding, and cardio-respiratory events from intravenous sedation). To better understand the follow-up procedure and to obtain a truly informed decision about CRC screening, we believe it is necessary to promote more balanced information rather than a one sided message, which states screening can only be universally beneficial [77,78].

Overall, the percentage of correct answers for knowledge of CRC risk factors was high. We suggest that Japanese people are highly aware of CRC as a result of 10-year national CRC screening program. Screening participants also appear to be relatively health-conscious and thus, reported a greater knowledge of CRC. Similar to reports from previous studies [29,31], few subjects are aware that increasing age is a risk factor for CRC. This lack of awareness may be explained by the suggestion that individuals do not conceptualize unmodifiable characteristics (e.g., gender and age) as risk factors [29]. Knowledge may be viewed as a distal predictor of intention, and in accordance with other research results [31]; our findings demonstrate that knowledge is positively associated with intention. As knowledge of risk factors for cancer has increased, the intention to undertake screening has increased [29]; educating public regarding the risk factors of CRC and increasing public awareness of CRC is required. A limitation of the current study was a restricted assess to knowledge of risk factors, thus further studies should be conducted to provide insight into the other domains of knowledge regarding CRC.

Perceived severity has a tendency to associate with intention, but failure to achieve statistical significance. Our finding is not consistent with results from previous studies that have reported that perceived susceptibility has an association with behavioral intention [33,36,44,50,67]. It is possible that perceived susceptibility might be indirectly connected to follow up intention mediated by the others variables [36,79]. Social support was not a significant predictor of intention in this study, and it may due to the relative crudeness of our measurement. Furthermore the sample in our investigation was homogenous, limiting the ability to detect significant associations between patients' characteristics and intention.

This study should be perceived as the first step concerning the investigation of patients' factors potentially associated with inadequate follow-up of abnormal FOBT screening. Our findings should be interpreted in light of some important limitations. Firstly, since this is a cross-sectional study, a strong inference about causality cannot be drawn. Secondly, intention was utilized as the outcome variable in the present study. While a considerable body of research supports the predictive validity of intentions for a variety of behaviors, prospective studies should be conducted to determine how well the variables identified here, including intention, and other variables serve to predict actual compliance with follow-up tests. Thirdly, since this sample was recruited from a single community the generalization of findings is limited. Fourthly, since this study examined patients' beliefs regarding CRC and follow-up under a potentially abnormal FOBT, combined with the limited sample size of patients who reported previous bowel examination was not possible, further research is required to embrace those patients. Finally, the schedule for this study did not allow time for a large pilot study sample to establish reliability and validity of perceived barriers scales, and a recommendation for further research would be for further analysis to be conducted correlating social-psychosocial variables and intention in one model simultaneously.

## Conclusion

Despite a number of limitations, this theory-driven research identified three factors that were significantly associated with intentions to undergo follow-up exams: perceived barriers, perceived benefits, and knowledge of CRC risk factors. Ours findings suggest that future interventions should focus on reducing modifiable barriers by clarifying misperceptions about follow-up, promoting the acceptance of CDE, addressing psychological distress, and making follow-up testing more convenient and accessible. Moreover, educating the public regarding the risk factors of CRC and increasing understanding of the benefits of follow-up is also important. We believe that further research is required to apply the findings from this study to develop appropriate information and effective methods of communication and to identify the best strategies for interventions.

## Competing interests

The author(s) declare that they have no competing interests.

## Authors' contributions

ZYF was involved in design, collecting data, analysis of data, interpretation and drafting manuscript. ST was involved in design, questionnaire development, analysis and interpretation of data. TM assisted with the study design, questionnaire development and interpretation of data. IT assisted with the study design and collecting data. KI supervised the entire project. All the authors have read and approved the final manuscript.

## Pre-publication history

The pre-publication history for this paper can be accessed here:



## Figures and Tables

**Table 1:** Characteristics of study sample (n = 288)

Variable	% or mean(SD)
Sociodemographic characteristics	
Age	
< 50	23.8
50–59	27.7
60–69	28.3
>=70	20.2
Gender	
Men	33.3
Marital status	
Married	89.0
Living arrangement	
Living alone	5.8
Spouse/partner only	37.5
With children	35.5
With parents (and children)	20.9
Education	
Less than high school	10.3
High school	62.1
Junior college/technical school	17.7
College degree or higher	9.9
Employment status	
Employed	60.8
Self-rated economic status	
Indigent/somewhat indigent	16.3
Average	69.1
Affluent/somewhat affluent	14.5
Personality	
Trait anxiety (20 – 80)	43.9(8.7)
Health status	
Subjective health	
Good/very good	31.6
Fair	55.6
Poor/very poor	12.8
Have chronic disease	
Yes	44.8
Have bowel symptoms	
Yes	53.8
Family history of CRC	
Yes	14.2
Past FOBT screening	
Yes	69.3

Note: CRC, colorectal cancer; FOBT, fecal occult blood test.

**Table 2:** Logistic regression of intention to adhere to recommendations for follow-up after an hypothetical abnormal FOBT result (n = 288)

			Bivariate analyses		Multivariate analyses	
				
		respondents	crude		adjusted^a^	
				
Variables	range or category	% or mean(SD)	OR(95%CI)	*p *value	OR(95%CI)	*p *value
Intention	high	74.7				
Perceived barriers						
Misperception regarding the necessity of follow-up						
CDE are not necessary unless symptomatic	1 – 5	1.8(1.2)	0.70(0.56–0.87)	0.002	0.67(0.53–0.85)	0.001
CDE are not necessary for my age	1 – 5	1.7(1.1)	0.64(0.51–0.81)	< 0.001	0.60(0.47–0.77)	< 0.001
Discomfort with the CDE procedure						
Having CDE would be embarrassing	1 – 5	2.8(1.5)	0.85(0.70–1.02)	0.072	0.76(0.62–0.94)	0.010
Bowel preparation for the CDE is too difficult	1 – 5	3.8(1.3)	1.11(0.90–1.37)	0.316	1.06(0.85–1.33)	0.589
Having CDE would be uncomfortable	1 – 5	3.6(1.4)	0.97(0.80–1.18)	0.763	0.91(0.73–1.13)	0.403
Having CDE would be painful	1 – 5	3.6(1.4)	0.98(0.81–1.20)	0.870	0.91(0.73–1.13)	0.410
Psychological costs						
I will feel uncomfortable talking about CRC	1 – 5	2.7(1.4)	0.84(0.69–1.02)	0.073	0.80(0.65–0.99)	0.037
I am afraid of finding cancer	1 – 5	3.5(1.4)	0.92(0.76–1.12)	0.435	0.86(0.72–1.09)	0.250
Practical barriers						
I am too busy to undertake follow-up examinations	1 – 5	1.8(1.1)	0.54(0.42–0.70)	< 0.001	0.48(0.36–0.63)	< 0.001
I have other things to do that are more important than undertaking follow-up examinations	1 – 5	1.4(0.9)	0.59(0.44–0.79)	< 0.001	0.56(0.41–0.76)	< 0.001
Having CDE would cost too much money	1 – 5	1.8(1.1)	0.75(0.59–0.95)	0.016	0.71(0.54–0.92)	0.009
Perceived benefits	6 – 30	25.0(5.5)	1.07(1.02–1.12)	0.008	1.06(1.02–1.12)	0.010
Perceived susceptibility	3 – 15	6.3(2.7)	1.02(0.92–1.12)	0.744	1.05(0.94–1.17)	0.377
Perceived severity	6 – 30	19.0(5.9)	1.04(1.00–1.09)	0.071	1.05(0.99–1.10)	0.094
Knowledge of CRC risk factors^b^						
Blood relative with CRC	correct	77.1	2.04(1.10–3.77)	0.023	2.06(1.07–4.12)	0.030
Low fiber diet	correct	91.5	2.49(1.04–5.96)	0.041	2.72(1.07–6.90)	0.035
High fat diet	correct	85.6	1.52(0.74–3.14)	0.258	1.33(0.60–2.95)	0.491
Increasing age	correct	45.8	1.20(0.69–2.07)	0.515	1.22(0.69–2.12)	0.486
Social support^c^						
Health care professionals	yes	55.9	1.12(0.65–1.92)	0.695	1.15(0.65–2.05)	0.633
Family members	yes	53.1	1.04(0.59–1.85)	0.884	0.96(0.53–1.75)	0.902

Note: OR: odds ratio; CI, confidence interval; CDE, complete diagnostic evaluation; CRC, colorectal cancer; FOBT, fecal occult blood test.

^a ^adjusted for age, gender, marital status, education, economic status, trait anxiety, bowel symptoms, family history of CRC, and past FOBT screening. ^b ^correct vs. else. ^c ^yes vs. not.

For the scales, the OR estimate the change in odds of intention that is associated with a change of one scale point.

The OR described the associated of each psychosocial variable and intention respectively.
